# Interpreting IGF-1 in children treated with recombinant growth hormone: challenges during early puberty

**DOI:** 10.3389/fendo.2024.1514935

**Published:** 2025-01-21

**Authors:** Helena-Jamin Ly, Carina Ankarberg-Lindgren, Hans Fors, Staffan Nilsson, Jovanna Dahlgren

**Affiliations:** ^1^ Department of Pediatrics, Göteborg Pediatric Growth Research Center (GP-GRC), Institute of Clinical Sciences, Sahlgrenska Academy, University of Gothenburg, Gothenburg, Sweden; ^2^ Department of Pediatric Endocrinology, Sahlgrenska University Hospital, Queen Silvia Children Hospital, Gothenburg, Sweden; ^3^ Department of Laboratory Medicine, Institute of Biomedicine, Sahlgrenska Academy, University of Gothenburg, Gothenburg, Sweden

**Keywords:** growth hormone dosing, growth hormone treatment, insulin-like growth factor-1, pubertal signs, sex steroid

## Abstract

**Objective:**

It can be challenging to determine the correct dosage of recombinant growth hormone (GH) in children with GH deficiency, leading to highly variable treatment responses. Insulin-like growth factor-1 (IGF-1) is a tool for monitoring GH treatment and dosing. However, IGF-1 levels depend on sex, age, and pubertal stage, amongst other factors, making its interpretation somewhat difficult. This study aimed to evaluate descriptively a group of 93 children treated per protocol with GH to assess the influence of pubertal signs and sex steroid levels on the interpretation of IGF-1.

**Methods:**

93 (67 boys and 26 girls) prepubertal children who participated in a previous GH treatment trial were included. Age, pubertal stage, weight, height, GH dose, and IGF-1 plasma concentrations were collected at least yearly from 2 years before pubertal start and 3 years after pubertal start. Levels of estradiol in girls and testosterone in boys were analyzed from previously collected frozen samples.

**Results:**

Nine of 58 (15.5%) estradiol samples in girls with Tanner breast stage 1 had pubertal levels of estradiol ≥25 pmol/L. For boys with testes size <4 mL, 24 out of the 153 (15.7%) testosterone samples were above the pubertal cut-off, ≥0.47 nmol/L. All the IGF-1 samples were divided into two groups based on an IGF-1 standard deviation score (SDS) of ≥2 or <2 SDS. The IGF-1 ≥2 SDS samples had a higher median (range) GH dose, 0.042 (0.02-0.10) mg/kg/day, compared with the IGF-1 <2 SDS samples, 0.038 (0.01-0.10) mg/kg/day, p<0.001. In the IGF-1 ≥2 SDS samples vs the IGF <2 SDS samples, estradiol levels were lower among girls, 13 (3-214) vs 102 (1-1070) pmol/L p<0.001, and testosterone levels were lower among boys, 0.35 (0.11-27.2) vs 6.9 (0.04-31.2) nmol/L p<0.001.

**Conclusion:**

Interpretation of IGF-1 near puberty is challenging due to the influence of sex steroids. Variations in sex steroid levels and pubertal status can lead to misleading interpretations and an overestimation of IGF-1 SDS. Establishing an IGF-1 reference range that includes sex steroid levels can improve its clinical use to monitor GH treatment.

## 1 Introduction

In children with growth hormone (GH) deficiency, response to recombinant GH treatment is highly variable due to many factors, making correct dosing challenging (1–4). International guidelines recommend weight- or body surface-based dosing because of a lack of evidence to support alternative dosing strategies (5). A potential dosing approach involves titration of GH to the serum levels of insulin-like growth factor-1 (IGF-1).

IGF-1, a biomarker of GH status, could guide GH treatment but has some limitations. Serum IGF-1, which is measured clinically, is mainly derived from hepatic production mediated by GH and is negatively affected by catabolic conditions (6), and inter-assay variability adds complexity to the interpretation (7). Moreover, there is considerable individual variability in IGF-1 levels. In a pediatric cohort with individuals at different stages of maturation, using IGF-1 as a biomarker poses even greater challenges as growth rates and adult height also depend on birth length, bone age maturation, and nutritional status (8).

Short-term height benefits of GH treatment have been shown with an IGF-1 target defined as a standard deviation score (SDS) of 0 or +2 compared to weight-based dosing. Increasing the dose of GH to reach higher IGF-1 targets has yielded enhanced growth responses (4, 9). In a randomized controlled trial, Cohen et al. showed that GH dosing targeted to IGF-1 0 SDS could be dose-sparing compared to conventional weight-based dosing and theoretically safer as high IGF-1 levels were avoided (10). However, an optimal target for long-term IGF-1 levels has not yet been established to optimize height outcomes and address safety and cost concerns, which would be needed to make a recommendation regarding IGF-1 titration in GH dosing.

Currently, the recommended use of IGF-1 is as a long-term safety marker for patients treated with GH. It is recommended that levels of IGF-1 should not exceed 2 SDS due to concerns about increasing the potential risk of adverse events (5). Epidemiological studies in adults have shown an association between IGF-1 levels in the upper quartile of the normal range and an increased risk of colorectal cancer, breast cancer, and prostate cancer, while IGF-1 levels in the lower quartile of the normal range have been linked to ischemic heart disease (11, 12). Short-term elevation of IGF-1 has occasionally been acceptable and has not led to severe adverse effects, although supraphysiological doses have been shown to accelerate bone maturation (13). The national Swedish guidelines from 2020 recommend a standard GH dose of 33µg/kg/day adjusted for IGF-1 with an upper limit below 2 SDS, whereas in the first two years of treatment, an IGF-1 up to 3 SDS can be short-term tolerated if necessary. Compliance with these guidelines requires a reliable IGF-1 reference range.

IGF-1 levels gradually increase during childhood and appear to peak in mid-puberty, with high levels occurring two to four years after the pubertal growth spurt (14). Levels of IGF-1 peak during puberty, indicating a link between IGF-1 and endogenous sex steroids and, hence, sexual maturation (14–16). It has been suggested that the GH endogenous production pattern is modulated by estradiol as previous studies have shown that estrogen receptor blockers and aromatase inhibitors in males tend to decrease GH and IGF-1 levels (17, 18). In our clinic, we have occasionally seen that children treated with GH during the early pubertal years have increases in IGF-1 despite having no clinical pubertal signs according to Tanner staging nor changes in the GH dose. We hypothesized that this pattern could be due to an early rise in sex steroid levels before the appearance of clinical pubertal signs.

Whether IGF-1 will be used for GH dose titration or treatment monitoring, the correct interpretation of the levels is important. The objective of the current study is to assess a well-defined group of peripubertal children with short stature receiving GH treatment to investigate how pubertal maturation and sex steroid levels may impact the interpretation of IGF-1 and the characteristics of the children with higher or lower serum IGF-1 SDS.

## 2 Materials and methods

### 2.1 Study population and design

A total of 98 children (72 boys and 26 girls) who previously participated in a GH dosing clinical trial (n = 33 with GH-deficiency; n = 65 with non-GH-deficiency based on both a provocation test and a spontaneous GH-secretion profile) were considered eligible for the study. The trial was conducted in Sweden at five pediatric clinics in Gothenburg, Umeå, Uppsala, Malmö, and Halmstad (Swedish study number NRA 6280003 and ClinicalTrials.gov identifier NCT02879747). See previous publication for more details about the cohort (19).

The time frame of our study spanned 2 years before pubertal start and 3 years after pubertal start, thus, up to 6 years of follow-up data were available. Pubertal starts were defined as Tanner breast stage >1 in girls, and at least one testis >3 mL in boys. We collected clinical data from a trial database, including age, pubertal stage, weight, height, GH dose, IGF-1, and IGF-1 SDS. Blood samples were taken at least yearly and stored in a freezer at -80 degrees Celsius. We assembled a set of frozen samples obtained in the morning between 0800-1000 am closest in time to clinical pubertal start, one year and two years prior, and one, two, and three years after the start of puberty. For the girls, we analyzed estradiol levels, and for the boys, testosterone levels (20). Five boys were excluded because of a lack of clinical data or lack of a series of stored blood samples. A total of 93 children were hence included (67 boys and 26 girls).

Of a total of 475 IGF-1 measurements, 31 were missing for the dates when data on sex steroids were available, and thus, these missing IGF-1 data were analyzed later from frozen samples. Weight and height data were collected +/- 30 days from IGF-1 and sex steroid sampling dates.

### 2.2 Hormonal analyses

Serum IGF-I concentrations were measured using a specific radioimmunoassay (RIA) (Mediagnost GmbH. Tübingen, Germany) (21). The total coefficient of variation (CV) was 18% at 40 µg/L and 11% at 225 µg/L. For IGF-1 analyses of the 31 frozen samples, we used the chemiluminescent immunoassay IDS iSYS (Immunodiagnostic Systems Holdings, Tyne and Wear, UK). The two methods correlate with r=0.98, and no conversion was needed.

Serum estradiol and testosterone levels were simultaneously determined by gas chromatography-tandem mass spectrometry (GC-MS/MS), as described in detail elsewhere (20). The lower limit of detection (LOD) for estradiol was 2 pmol/L, and for testosterone, 0.1 nmol/L. Total CV for estradiol was 19% at 8 pmol/L and 6% at ≥36 pmol/L, and for testosterone, 16% at 0.3 nmol/L and <10% at >1.5 nmol/L.

Biological reference range for serum estradiol and testosterone in children have previously been published (22). Replacing (extraction-) RIAs with the newer GC-MS/MS provides a high degree of agreement between the methods (20), and therefore, previously established reference ranges could be adopted. In girls with Tanner breast stage 2, 90% of the serum estradiol concentrations ranged between 7 and 77 pmol/L. However, estradiol concentrations ≤24 pmol/L were seen in both prepubertal girls and in girls with Tanner breast stage 2. Therefore, we assigned an estradiol concentration of ≥25 pmol/L as a cut-off value for pubertal levels in girls. Following similar reasoning, we assigned a testosterone concentration of ≥0.47 nmol/L as a cut-off value for pubertal levels in boys. For evaluation purposes, the results of < LOD were assigned the values of LOD/2.

IGF-1 levels were converted to SDS values according to the reference range of Löfqvist et al. (15). The reference range is sex- and age-specific and is also divided into four pubertal stages: prepubertal, early puberty, mid-puberty, and late puberty.

BMI SDS was calculated according to Karlberg et al. (23).

### 2.3 Statistical methods

Data are expressed as medians or means (range). Linear mixed effect models were used for the comparison of sample groups. P-values <0.05 are considered statistically significant.

### 2.4 Ethics

The research protocol was approved by the local Research Ethics Committee with ref nr 320-03, 449-16 and the Medical Product Agency of Sweden study no., NRA 6280003. Parents signed informed consent, and assent was obtained from the child when age-appropriate. The study was performed according to the Helsinki Declaration.

## 3 Results

### 3.1 Patient characteristics and pubertal start

During the study period, the average number of samples analyzed for sex steroids per child was 4.8 (2-6) for girls and 5.2 (3-6) for boys. In girls with a clinical pubertal stage of Tanner breast stage 1, a total of 58 samples were analyzed to quantify estradiol concentrations, and for boys with testes size <4 mL, a total of 153 samples were analyzed to quantify testosterone concentrations. Characteristics of the children included in the study are described in **Table 1**. Puberty started at a normal age, at a median of 11.0 (9.5-12.5) years for girls and 12.5 (10.4-14.7) years for boys. At the start of puberty, IGF-I values were increased for most children, as expected.

**Table 1:** Patient characteristics.

Variables	Girls (n=26)		Boys (n=67)	
	n=	Median (range)	n=	Median (range)
Age at first follow-up	26	8.9 (7.2-11.8)	67	10.7 (6.6-14.7)
Age at the start of clinical puberty (Tanner II)	26	11.0 (9.5-12.5)	66	12.6 (10.4-14.7)
GH dose (mg/kg/d) at each point	118	0.040 (0.02-0.10)	332	0.039 (0.01-0.10)
Estradiol in clinically prepubertal girls (pmol/L)	58	9 (0-147)		
Testosterone in clinically prepubertal boys (nmol/L)			153	0.28 (0-13.3)
IGF-1 SDS (µg/L)	126	1.4 (-2.0-4.1)	349	1.4 (-4.2-4.1)

GH, growth hormone; IGF-I, insulin-like growth factor 1; SDS, standard deviation score.

### 3.2 Sex steroids and IGF-1 SDS

**Figure 1:**
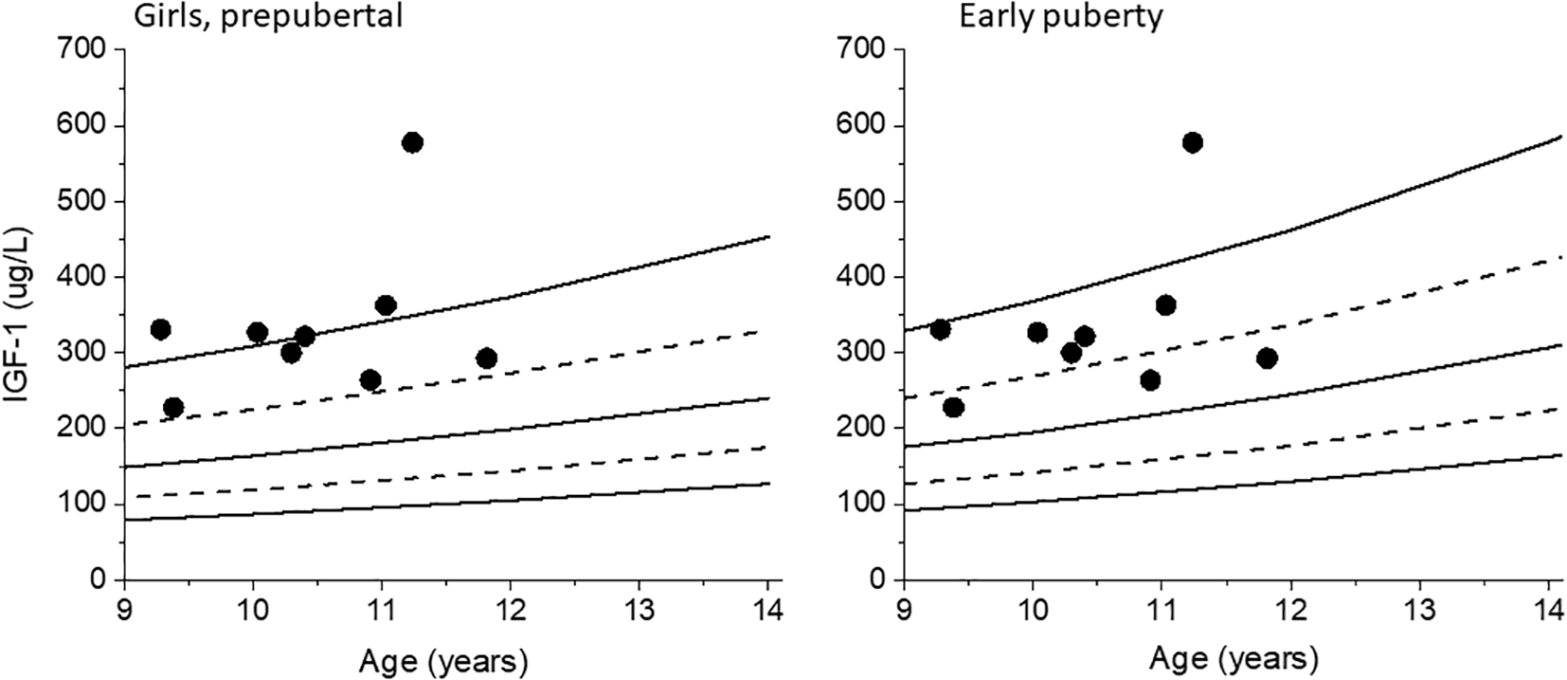
IGF-1 values for the girls with Tanner breast stage 1 but with pubertal levels of estradiol are plotted against the prepubertal reference range for girls, left. The same IGF-1 values are plotted against the early puberty reference range, right. Dashed lines +/- 1 SDS, solid lines mean and +/- 2 SDS.

The hormonal start of puberty in girls was defined using an estradiol concentration of ≥25 pmol/L as a cut-off value. Nine of 58 (15.5%) samples from girls with clinically-defined Tanner breast stage 1 had levels above this cut-off. When recalculating IGF-1 SDS for these samples using the IGF-1 reference range corresponding to early puberty instead of prepuberty, the IGF-1 SDS decreased from a median of 2.04 (1.29-3.62) to 1.44 (0.63-2.98), p<0.05. Four out of nine IGF-1 SDS levels decreased below the clinical dose-adjusting upper limit of 2 SDS recommended by guidelines (5). See **Figure 1**.

**Figure 2:**
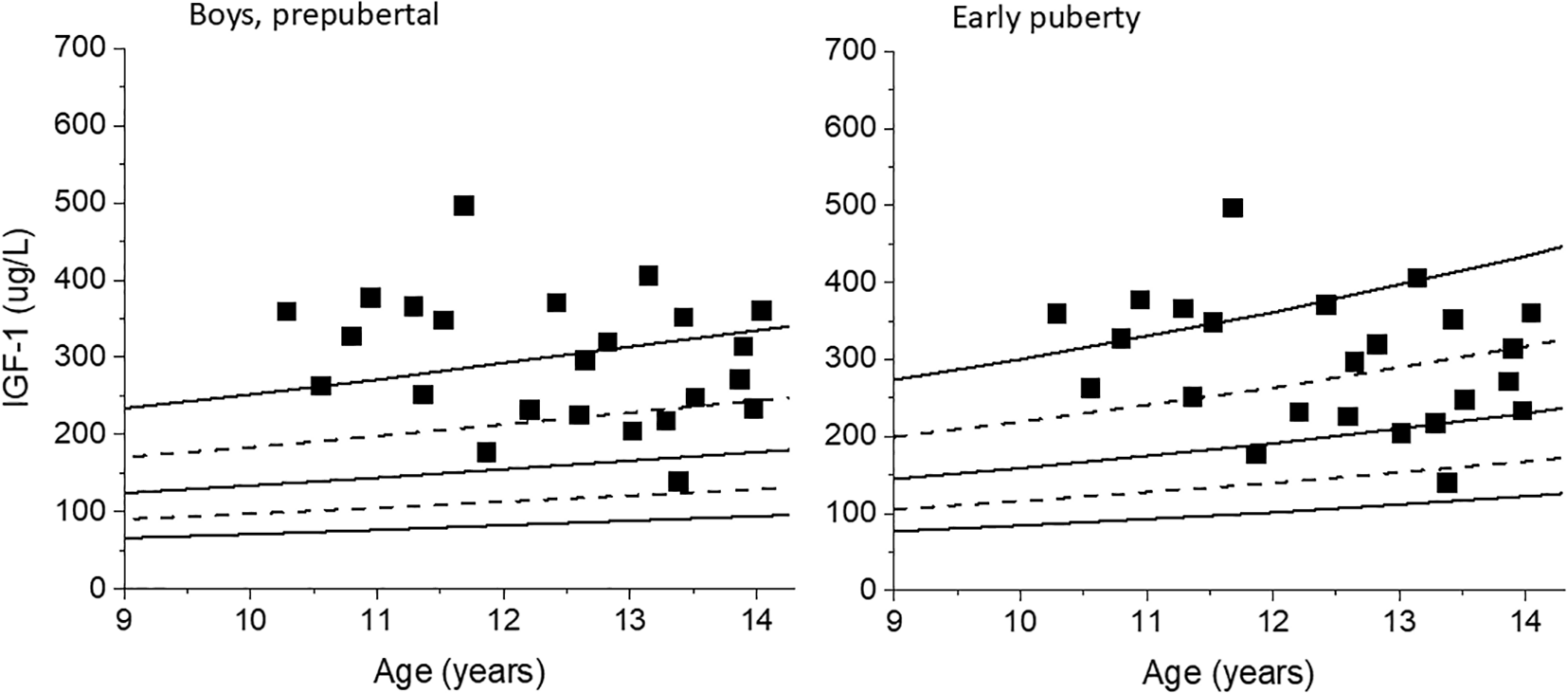
IGF-1 values for boys with testes size <4 mL but with pubertal levels of testosterone are plotted against the prepubertal reference range for boys, left. The same IGF-1 values are plotted against the early puberty reference range, right.

For the boys with testes size <4 mL, 24 out of the 153 samples (15.7%) had pubertal levels of testosterone above the cut-off value of ≥0.47 nmol/L. Of these 24 samples with pubertal testosterone levels, 16 coincided with a testes size of 3 mL. Recalculating IGF-1 SDS for these 24 samples using the early puberty reference range instead of the prepubertal reference range decreased the median IGF-1 SDS from 1.96 (-0.66-3.75) to 1.29 (-1.42-3.10), p=0.02. Five out of the 24 IGF-1 SDS levels decreased below the clinical dose- adjusting upper limit of 2 SDS. See **Figure 2**.

### 3.3 Comparing IGF-1 groups

Assessing all the IGF-1 data independent of pubertal staging, 166 of 475 (35%) samples for IGF-1 were ≥2 SDS, and the rest were <2 SDS. Characteristics of children with IGF-1 ≥2 SDS and <2 SDS were compared, and significant differences were found, see **Table 2**. The IGF-1 ≥2 SDS samples had a higher median GH dose, compared with the IGF-1 <2 SDS samples. In the IGF-1 ≥2 SDS samples vs the IGF <2 SDS samples, estradiol levels were lower among girls, and testosterone levels were lower among boys. Further stratification of the data by diagnosis yielded consistent results for the non-GHD, with same significant differences observed as prior to stratification. The GHD group was very small and hence the results not conclusive with **Table 2** (see **Supplementary Table S1**).

**Table 2:** Characteristics of the children at samples of either high or low IGF-1 SDS.

Variables		IGF-1 ≥2 SDS		IGF-1 <2 SDS	
	n=	Median (range)	n=	Median (range)	p-value
Mean GH dose (mg/kg/day)	140	0.042 (0.02-0.10)	283	0.038 (0.01-0.10)	p<0.001
Mean GH dose (mg/m^2^)	140	1.24 (0.49-2.94)	283	1.15 (0.42-3.22)	p=0.08
BMI (kg/m^2^)	153	17.5 (13.5-26.1)	319	17.9 (13.0-27.7)	p<0.001
BMI (SDS)	151	-0.18 (-4.16-2.49)	318	0.10 (-2.65-2.26)	p=0.23
Estradiol in girls (pmol/L)	42	13 (3-214)	83	102 (1-1070)	p<0.001
Testosterone in boys (nmol/L)	236	0.35 (0.11-27.2)	112	6.9 (0.04-31.2)	p<0.001

BMI, body mass index; GH, growth hormone; IGF-I, insulin-like growth factor 1; SDS, standard deviation score.

## 4 Discussion

GH dosing is challenging due to individual variability in sensitivity. Based on our clinical experience, we hypothesized that children treated with GH during the early pubertal years could have increases in IGF-1 due to an early rise in sex steroid levels before the appearance of clinical pubertal signs. The present retrospective study showed that as many as 15% of children undergoing GH treatment had pubertal levels of sex steroids in the absence of pubertal clinical signs. This proportion exceeds the expected 5% corresponding to children in the 90^th^ percentile of the pubertal cut-off values for the sex steroids. The median estradiol for the clinically prepubertal girls in the cohort was 9 pmol/L and for the prepubertal boys 0.28 nmol/L, however one girl had a surprisingly high level of estradiol of 147 pmol/L, and one boy had surprisingly high testosterone of 13.3 nmol/L. Both samples were reanalyzed showing the same results. The girl attained menarche at the age 12 and the boy only attained 12 mL in testicle size by the age 18.

In clinical practice, IGF-1 titration is one of several tools to facilitate GH dosing, given that there is a reliable reference range. Some clinics use reference ranges that are age- and sex-dependent but lack pubertal staging. In a GH-naïve cohort of peripubertal children, Inoue-Lima et al. showed that IGF-1 reference ranges adjusted for the pubertal stage have the best positive predictive value of GH deficiency (24). The present study used the IGF-1 reference range by Löfqvist et al. (15), which includes age and gender, but also clinical pubertal staging expressing IGF-1 levels as SDS, which we believe is needed to accurately interpret IGF-1 SDS as sex steroids are such an important factor affecting IGF-1 levels.

An upper limit of 2 SDS for IGF-1 levels is often recommended in international guidelines when treating children with GH for safety reasons (5). In our study, nine of the 58 samples in clinically prepubertal girls had pubertal levels of estradiol, and when IGF-1 SDS was recalculated using the pubertal reference range, 4 had IGF-1 <2 SDS. Similarly, five out of 24 pubertal samples in clinically prepubertal boys had IGF-1 <2 SDS. In these patients, an unnecessary decrease in GH dose would have been applied outside the clinical trial according to guidelines.

When we divided the cohort based on IGF-1 SDS values of ≥2 or <2, we found a remarkable difference in sex steroid expression. Girls with IGF-1 ≥2 SDS had much lower estradiol levels than those with <2 SDS (13 [3-214] pmol/L vs 102 [1-1070] pmol/L, p<0.001). In a similar vein, boys with IGF-1 ≥2 SDS had lower testosterone levels than those with <2 SDS (0.35 [0.11-27.2] nmol/L vs 6.9 [0.04-31.2] nmol/L, p<0.001). These results indicate that children in the early pubertal years tend to be labeled with higher IGF-1 SDS, possibly due to an overestimation of IGF-1 SDS by the reference range used clinically. This finding aligns well with what has been described clinically, where a rise in IGF-1 SDS is seen during the early pubertal years in clinically prepubertal children. However when stratified by diagnosis, the GHD group showed early pubertal estradiol levels for both high and low IGF-1 SDS, possibly due to the small number of data in each group.

In further analyzing the differences between the groups with higher and lower IGF-1 SDS, we could see that the IGF-1 ≥2 SDS samples corresponded to a significantly higher GH dose per body weight, as expected. However, this was not the case when expressing the GH dose per body surface area and is in line with the finding of a slightly lower BMI in the IGF-1 ≥2 SDS samples, 17.5 (13.5-26.1) vs 17.9 (13.0-27.7) kg/m^2^ p<0.001.

While most recommendations for GH dosing are based on body weight, some studies have evaluated GH dosing by IGF-1 titration. Cohen et al. showed in a randomized controlled trial that titrating GH doses to achieve higher IGF-1 targets resulted in augmented growth responses compared to conventional dosing in pre-pubertal children with short stature, although using higher GH doses (9). Titrating IGF-1 targeted to the age- and gender-adjusted mean seemed to be dose-sparing and theoretically safer as higher IGF-1 levels were avoided (10). This shows the benefits of IGF-1 titration and we believe that by addressing the pitfalls of IGF-1 interpretation we can come closer to understanding IGF-1 and possibly have greater use to it clinically.

However, in patients with relative IGF-1 resistance, such as the case with children born small for gestational age or girls with Turner syndrome, seem not to benefit from IGF-I titration within the normal range (25, 26). These children would have required higher GH doses and had higher IGF-1 levels during GH treatment to achieve an adult height within the normal range.

A strength of the study is that the cohort is from a previous randomized controlled clinical study, and thus, the data are controlled and reliable, although the size of the cohort is limited. Moreover, we used stringent cut-off levels for the sex steroid levels to ensure that children had definitively reached puberty clinically as well as biochemically, albeit with a possibility of missing a few pubertal children with low sex steroid levels. Adherence data were collected thoroughly during the prepubertal and early pubertal years by counting empty syringes, although digital data from accurate devices were lacking. Moreover, the longitudinal approach with sampling at least every twelve months for six consecutive years is also an advantage in discovering changes over time. GH dosing was per protocol and, thus, was not altered depending on the IGF-1 levels, resulting in IGF-1 levels that reached 35% above international recommendations of +2 SDS. This made it possible for us to analyze and identify the differences between children with lower and higher IGF-1 SDS in the study cohort. Although we believe that IGF-1 SDS is overestimated in some children in their early pubertal years, this notion is hard to prove. To our knowledge, there is no IGF-1 reference range based on sex steroid levels rather than clinical pubertal signs, apart from sex and age.

Based on the results of this study, we encourage further analyses of sex steroid levels when an increase in IGF-1 SDS is detected in prepubertal children where GH dosing has not been altered. If the sex steroid levels are above the pubertal cut-off values, we believe it is beneficial to use the early puberty reference ranges for these children rather than decreasing the GH dose and practicing close monitoring. We are convinced that IGF-1 is an important and useful tool in GH dosing, but more accurate reference ranges, preferably those including sex steroid levels, are needed. There is also a need to conduct further research on IGF-1 to investigate factors that influence levels during GH treatment to be able to fully utilize its potential as a useful biomarker.

## 5 Conclusion

This work highlights challenges in interpreting IGF-1 levels during the early pubertal years due to the influence of sex steroids. A substantial number of patients undergoing GH treatment may have IGF-1 SDS levels that are overestimated around the onset of puberty. Based on the present results, we stress the importance of sex steroid analyses in IGF-1 reference range to be able to better interpret results in peripubertal children.

## Data Availability

The raw data supporting the conclusions of this article will be made available by the authors, without undue reservation.
