# Supplementary material for: Interpreting IGF-1 in children treated with recombinant growth hormone: challenges during early puberty
Source: Front Endocrinol (Lausanne). 2025 Jan 21;15:1514935. doi: 10.3389/fendo.2024.1514935 (PMC11790427; doi:10.3389/fendo.2024.1514935)
Supplement: Supplementary file 1 [file Table1.docx]

|  |  |  |  |  | |  | |  |  |  |
| --- | --- | --- | --- | --- | --- | --- | --- | --- | --- | --- |
|  |  |  |  |  |  | |  | |  |  |

Supplementary Table 1. Characteristics of the children at samples of either high or low IGF-1 SDS stratified by diagnosis.

| Non-GHD |  |  |  |  |  |
| --- | --- | --- | --- | --- | --- |
| **Variables** |  | **IGF-1 ≥2 SDS** |  | **IGF-1 SDS<2** |  |
|  | n= | Median (range) | n= | Median (range) | p-value |
| Mean GH dose (mg/kg/day) | 101 | 0.040 (0.02-0.10) | 197 | 0.038 (0.01-0.10) | p<0.001 |
| Mean GH dose (mg/m^2^) | 101 | 1.22 (0.48-2.94) | 197 | 1.17 (0.42-2.87) | p<0.001 |
| BMI (kg/m2) | 101 | 17.3 (14.1-24.1) | 210 | 17.9 (13.4-25.2) | p<0.001 |
| BMI (SDS) | 101 | 0.11 (-2.65-2.26) | 210 | -0.18 (-4.16-2.49) | p=0.055 |
| Estradiol in girls (pmol/L) | 25 | 11 (3-214) | 64 | 112 (1-1070) | p<0.001 |
| Testosterone in boys (nmol/L) | 77 | 0.42 (0.11-27.2) | 147 | 8.0 (0.05-29.3) | p<0.001 |
|  |  |  |  |  |  |
| GHD |  |  |  |  |  |
| **Variables** |  | **IGF-1 ≥2 SDS** |  | **IGF-1 SDS<2** |  |
|  | n= | Median (range) | n= | Median (range) | p-value |
| Mean GH dose (mg/kg/day) | 52 | 0.042 (0.02-0.10) | 101 | 0.038 (0.01-0.10) | p<0.001 |
| Mean GH dose (mg/m^2^) | 52 | 1.24 (0.62-2.90) | 101 | 1.14 (0.49-3.22) | p<0.001 |
| BMI (kg/m2) | 52 | 18.0 (13.5-26.1) | 109 | 17.9 (13.0-27.7) | p<0.001 |
| BMI (SDS) | 52 | 0.09 (-2.10-2.00) | 109 | -0.20 (-3.07-2.49) | p=0.773 |
| Estradiol in girls (pmol/L) | 17 | 20 (3-147) | 20 | 11 (1-185) | p<0.001 |
| Testosterone in boys (nmol/L) | 35 | 0.29 (0.11-15.0) | 90 | 5.45 (0.04-31.2) | p<0.001 |

BMI= body mass index, GH=growth hormone, GHD=growth hormone deficient, IGF-I=insulin-like growth factor 1, SDS=standard deviation score.
